# Is the way we’re dieting wrong?

**DOI:** 10.1186/s13073-016-0266-3

**Published:** 2016-01-21

**Authors:** Marc-Emmanuel Dumas

**Affiliations:** Section of Biomolecular Medicine, Division of Computational and Systems Medicine, Department of Surgery and Cancer, Imperial College London, Sir Alexander Fleming Building, Exhibition Road, South Kensington, London, SW7 2AZ UK

## Abstract

Progress in personalized medicine is now being translated to personalized nutrition. A recent proof-of-concept study shows that the increase in blood glucose levels after a meal is highly variable between individuals, but can be predicted by using a computational model that combines information from gut microbiome profiles and dietary questionnaires. This study raises questions about the usefulness of universal diet recommendations, and suggests we might need to move on to personalized diets.

## Metabolic syndrome and nutrition

More than a third of adults in the Western world who are over 20 years old have the metabolic syndrome [1]. The clinical definition of the metabolic syndrome varies but it typically includes at least three of the following symptoms: obesity, raised blood glucose levels, increased blood cholesterol levels and increased blood pressure, which together increase the risk of developing type 2 diabetes and cardiovascular disease. The common denominator behind the metabolic syndrome is insulin resistance, that is, a lack of sensitivity of peripheral organs to insulin, which has emerged as the root mechanism explaining the occurrence of these disorders.

After eating a meal, blood sugar levels increase in response to the absorption of digested nutrients. This postprandial glycemic increase (that is, the postprandial glycemic response (PPGR)) triggers secretion of insulin by pancreatic β cells. Increased levels of circulating insulin result in glucose uptake by peripheral organs, such as the liver, muscles and adipose tissue, and a return of blood glucose levels towards their normal physiological concentrations. Insulin resistance reduces the ability of the body to regulate blood glucose levels, which results in an increased PPGR despite increased insulin secretion. Insulin resistance and an increased PPGR are major risk factors for developing type 2 diabetes. In a recent study published in *Cell*, the teams of Eran Elinav and Eran Segal developed a personalized nutrition approach to predict PPGRs [2].

The metabolic syndrome has devastating consequences in economic and public health and in the quality of life of patients. Pharmacological approaches and surgical interventions such as bariatric surgery are effective in improving glycemic control and reducing weight. In particular, bariatric surgery remains the only effective way to cure type 2 diabetes. Despite the efficacy of these strategies, stomach stapling surgery has associated risks, just as taking pills for weight loss.

Dietary interventions are the easiest changes to implement in patients with the metabolic syndrome and offer a key lifestyle alternative to medication and surgery that has minimal adverse effects. These diets tend to follow universal guidelines, which recommend limiting the number of ingested calories while reducing fat and carbohydrate intake. These diets typically result in weight loss and improvement of glycemic control (reduction of PPGRs), but predicting which patients will respond to a particular diet is hard. Prediction of metabolic health in general, and PPGRs in particular, has remained empirical; genetics only explains a small amount of the variation in these factors and there is currently no effective way of predicting how each individual patient will respond to a particular diet.

## The emerging role of the microbiome

Our gut bacteria, collectively known as our gut microbiome, have a massive role in influencing the development of diabetes and obesity. The gut microbiome is now recognized as a key driver of inter-individual variation in the likelihood of developing obesity and diabetes. Individual microbiomes tend to cluster into enterotypes, which are communities within a statistical continuum that tend to be dominated by one phylum. Enterotypes are independent of age, sex and geographical location. Long-term dietary habits tend to influence these enterotypes. High intake of animal protein and fat, in particular, favors the growth of *Bacteroides*, whereas carbohydrate intake promotes growth of *Prevotella* species [3]. Several studies have demonstrated that high ecological diversity of gut microbes is associated with good health, which is presumably due to an increase in the diversity of bacterial functions. Owing to progress in sequencing technologies, it is now possible to measure almost every microbial gene in the microbiome and even a simple measure such as counting the total number of genes (microbial gene richness) shows an intriguing relationship of this number with metabolic health. Patients with a low microbial gene count tend to have more severe obesity, have more inflammation and gain more weight than patients with a high microbial gene count [4]. Patients with a low microbial gene count also respond better to dietary intervention than patients with a high microbial gene count [5]. Hence, the microbiome is now emerging as both a biomarker and an actionable target responding to dietary intervention in personalized medicine.

## From personalized medicine to personalized nutrition

A recent article by Zeevi et al. combines longitudinal monitoring of PPGRs by wearable continuous glucose monitors, microbiome profile data and clinical information in a large cohort. The findings show that although individual PPGRs are hypervariable, they are predictable by “big data” strategies, and suggest that personalized diets might be more successful than universal diets in controlling PPGRs. The researchers integrated various data types, including information on dietary intakes, anthropometric measurements, physical activity, sleep–wake cycles, high-resolution long-term blood glucose monitoring and fecal metagenomics in a cohort of healthy and pre-diabetic volunteers [2]. The 800 volunteers self-reported nearly 10 million calories consumed over nearly 47 thousand meals and automatically captured 1.5 million glucose measurements using ergonomic and minimally invasive continuous glucose monitors. In the first part of the study, Zeevi et al. established that PPGRs are highly variable, which suggests that universal dietary recommendations might have limited utility.

Having demonstrated the hypervariable nature of PPGRs, Zeevi and colleagues then analyzed individual anthropometric measurements, activity parameters and microbiome profiles to predict these responses. The researchers devised a machine-learning algorithm that is based on a gradient-boosting regression methodology, in which thousands of decision trees were derived to optimize the predictive value of the overall model. Careful calibration, with participants eating standardized meals, enabled the machine-learning algorithm to make precise predictions for real-life meals by bringing extra noise and coarse-grained resolution to the predictions, which are critical factors for modeling the human element in clinical studies. The investigators trained their model in the cohort of 800 individuals by using leave-one-out cross-validation and validated the model against an independent cohort of 100 volunteers.

Finally, Zeevi and colleagues used their gradient-boosting regression approach to design personally tailored dietary interventions, aiming at improving PPGRs in a two-arm blinded randomized controlled trial. Following the acquisition of baseline data, use of the machine-learning algorithm improved PPGRs as well as expert-based diet selection approaches.

## Key findings and significance of the study

The novel approach introduced by Zeevi et al. makes it possible to tailor diets to each individual. Although there was a high level of interpersonal variation among PPGRs, these were reproducible per person and per standardized meal and were associated with known risk factors. PPGR variability was also associated with clinical markers of diabetes (glycated hemoglobin levels), obesity (body mass index), liver function (alanine aminotransferase levels), inflammation (C-reactive protein levels), and, most importantly, with microbiome profiles. The bacterial taxa and microbial functions underlying these predictions were highly consistent with previous reports in the field, which further supports the robustness of the approach.

This work is an important proof-of-concept study that incorporates the contribution of the microbiome in the prediction of dietary responses. The approach is part of a new translational aspect of microbiome research. Other studies have pioneered the use of genome-scale modeling of microbiome responses to dietary intervention, which predicted short-chain fatty acid and amino acid variation in blood [6]. The combination of large-scale volunteer recruitment with longitudinal phenotyping depth enabled the machine-learning algorithm to outperform current models and expert-based predictions, making this study a cornerstone in the field of personalized nutrition. This study further supports the role of the microbiome as a prodromal marker—i.e., an early predictive marker—in pathophysiology. From a computational medicine angle, the study highlights the power of harnessing deep, longitudinal phenotyping by ‘big data’ strategies, which is critical to crack the code of complex individual response patterns and achieve individual predictions.

## Remaining challenges and future directions

This visionary study will undoubtedly influence the setup of nutritional interventions for the metabolic syndrome, but many questions remain unanswered. Will personalized diets based on big data predictions outweigh surgical and pharmacological interventions? Can metagenomic profiles and big data be used to gain a deeper understanding of the roles played by the microbiome in metabolic diseases? By adding more constraints to the machine-learning algorithm, is it possible to improve PPGRs whilst reducing aortic plaque formation or systemic inflammation?

This work clearly opens up new perspectives in the improvement of tailored dieting strategies, but a remaining challenge is increasing the availability of the enabling technology and the assessment of this machine-learning strategy in multicentric studies in various populations. Nonetheless, deploying these deep phenotyping and metagenomic strategies would certainly help patients with impaired glucose tolerance and obesity to have truly personalized diets. The ability to generate a personal baseline database including anthropometric measurements, blood test data, microbiome profiles and dietary reports obtained with smartphone-based apps is a powerful tool in terms of public health, patient engagement and patient outreach.

This work illustrates the global push for long-term phenotyping [7], metabolomics-assisted decision-making in surgical environments [8] and predicting the outcomes of toxicological interventions [9]. We are witnessing the marriage of deep metagenomics with wearable technology in personalized nutrition. This methodology is likely to affect patient handling in nutritional interventions, with diet recommendations probably shifting from universal guidelines, often criticized, to personalized diets. For optimal diets, bring on the personalized data deluge!

## Abbreviation

PPGR: postprandial glycemic response
